# A literature review of microvascular proliferation in arteriovenous malformations of skin and soft tissue

**Published:** 2021-07-30

**Authors:** Amalia Mulia Utami, Siham Azahaf, Onno J. de Boer, Chantal M. A. M. van der Horst, Lorine B. Meijer-Jorna, Allard C. van der Wal

**Affiliations:** ^1^Department of Pathology, Amsterdam University Medical Center, University of Amsterdam, Amsterdam, The Netherlands; ^2^Department of Pathology Anatomy, Faculty of Medicine, Universitas Hasanuddin, Makassar, Indonesia; ^3^Amsterdam University Medical Center, Vrije University, Amsterdam, The Netherlands; ^4^Department of Plastic Surgery, Amsterdam University Medical Center-location AMC, University of Amsterdam, Amsterdam, The Netherlands; ^5^Symbiant Pathology Expert Center, NWZ- Noordwest Ziekenhuisgroep, Alkmaar, The Netherlands

**Keywords:** arteriovenous malformation, vascular malformation, angiogenesis, microvascular proliferation

## Abstract

**Background and Aim:**

Arteriovenous malformations (AVM) are defined as being quiescent vascular masses composed of mature vessels. However, recent studies reported areas of microvascular proliferation (MVP) in AVM, indicating a process of angiogenesis. As this finding questions the previous definition, the primary objective of this review was to evaluate whether angiogenesis occurs in vascular malformations of skin and soft tissue, and second, to identify potential factors involved in MVP.

**Method:**

Due to the multifaceted nature of this subject, a hermeneutic methodology was used to select articles that were likely to provide a deeper understanding of MVP in vascular malformations. Through citation tracking and database searching in PubMed and Web of Science, relevant articles were identified. All study designs concerning occurrence of MVP in AVM of skin and soft tissue in all age groups were included in the study. The Newcastle-Ottawa scale was used for quality assessment.

**Results:**

16 studies were included in this review which reported occurrence of MVP areas in between the otherwise mature vessels of vascular malformations. In these studies, angiogenesis was reported only in AVM-type of vascular malformations. Increased levels of pro-angiogenic factors were also reported and proliferation was found most prominently during adolescence. Finally, several types of hormone receptors also have been described in tissues of AVM.

**Conclusion:**

Overall, the reviewed data support occurrence of active angiogenesis, highlighted by the presence of MVP in the arteriovenous type of vascular malformations, and a possible concurrent lesion progression towards a higher Schobinger stage of clinical severity. The relative scarcity of data at present implies that further research is required to elucidate the nature of MVP in AVM, which could have implications for developing targeted pharmacotherapy.

**Relevance for Patients:**

Active angiogenesis caused by MVP in AVM patients is known to be correlating to clinical symptoms and contributing to the progression of the disease, recurrence rate, and patient’s quality of life.

## 1. Introduction

Vascular anomalies are usually mass forming lesions of the vasculature that may affect capillaries, arteries, veins, and lymphatics, either single or in combinations. Despite being benign lesions in most of the cases, vascular anomalies can cause various serious complications depending on tissue type, location, and extent of the mass. Mulliken and Glowacki developed the first classification in 1982, and categorized vascular anomalies in vascular tumors and congenital vascular malformations, based on increased endothelial cells (EC) turnover in vascular tumors and a slowly progressive growth rate in the congenital malformations [1]. A revised and expanded version of the classification was established in 1996 by the International Society for the Study of Vascular Anomalies (ISSVA) [2]. This differentiation has certainly improved clinical recognition, yet identification of vascular malformations remained problematic.

According to a study performed by Greene *et al*. in 2011, a correct diagnosis of the type of vascular anomaly occurred in only 53% of the 5621 referral cases, of which 45.6% were vascular malformations and 70.4% of vascular tumors [3]. To improve diagnostic discrimination between the various subtypes of vascular anomalies, proper knowledge on the pathogenesis may be of great clinical value.

In 2006, areas of microvascular proliferation (MVP) were identified in a subgroup of vascular malformations, which appeared to be mostly high-flow arteriovenous malformations (AVM) of skin and soft tissue [4]. This finding challenged the definition of AVM as being quiescent, non-proliferative lesions and suggested a possible role for angiogenesis in the expansion of these lesions. AVM have an absent capillary bed between the arterial and venous component of the lesion, which results in so called “high-flow lesions” that may affect skin, soft tissue, and viscera. AVM (and other types of malformations) occur also in the brains, but these are not included in the ISSVA classification. Compared with purely venous, lymphatic or capillary malformations, AVM are potentially the most dangerous type of vascular malformations clinically, and are the most difficult to treat [5]. Collateralization, thickening of adjacent vessels and dilatation of the vessels are mechanisms considered to explain the enlargement of AVM [6]. Progression of clinical symptoms of AVM can be evaluated in the Schobinger’s clinical classification of AVM symptomatology (Table 1) [7].

**Table 1:** Schobinger’s clinical classification of AVM symptomatology [6]

Stage	Clinical findings
I (Quiescence)	Warm, pink-blue shunting on Doppler
II (Expansion)	Enlargement, pulsation, thrill, bruit, and tortuous veins
III (Destruction)	Dystrophic skin changes, ulceration, bleeding, and pain
IV (Decompensation)	Cardiac failure

**Figure 1:**
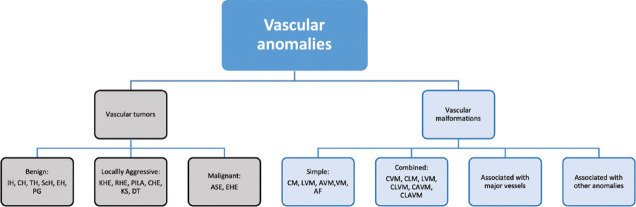
Simplified version, adapted from the ISSVA classification 2018 [9]

In 2014, a revised ISSVA classification was established, and more recently updated in 2016 and 2018 [8,9] due to ongoing advances in knowledge on the biological behavior, histopathology and underlying genetics of vascular anomalies. Although this classification still uses the dichotomous discrimination between vascular tumors and vascular malformations, subcategories are added to the group of vascular malformations: (1) Simple, (2) combined, (3) associated with major vessels, and (4) associated with other anomalies (syndromic lesions). According to the ISSVA classification, AVM can manifest as sporadic lesions, in patients with Hereditary hemorrhagic telangiectasia (HHT) and in patients with capillary AVM (CAVM) that are associated with Ras GTPase-activating protein 1 (RASA-1) mutation. Moreover, AVM can occur also in combination with other types of vascular malformations [10]. Lesions in which a clear diagnosis cannot (yet) be made are categorized as “provisionally unclassified vascular anomalies” [8]. However, the involvement of mass forming MVP is not considered in the expansion of AVM, and vascular malformations are still described by definition as: “A heterogeneous group of lesions that demonstrate cellular turnover without true proliferation, generally growing commensurate with the patient” [8]. A simplified version, adapted from ISSVA 2018 classification is shown in Figure 1.

A case study by Redondo *et al*. [11] further questions the current definition of vascular malformations. The authors reported extensive growth of AVM located in the trunk of a 51-years old man, showing the destructive consequences of histologically proven vascular proliferation. Moreover, serum levels of angiogenic factors were increased compared to control tissue. Eventually, the patient died due to multi-organ and renal failure.This case study serves also to demonstrate the importance of understanding angiogenesis in AVM, hence, to consider targeted anti-angiogenic therapy. Therefore, this review aims to evaluate whether angiogenesis, resulting in MVP, is involved in growth of congenital vascular malformations, and specifically which histological types of lesions involved, for which purpose we focused on skin and soft tissue lesions. Second, we tried to identify which factors could be involved in the process of angiogenesis.

## 2. Method

**Figure 2:**
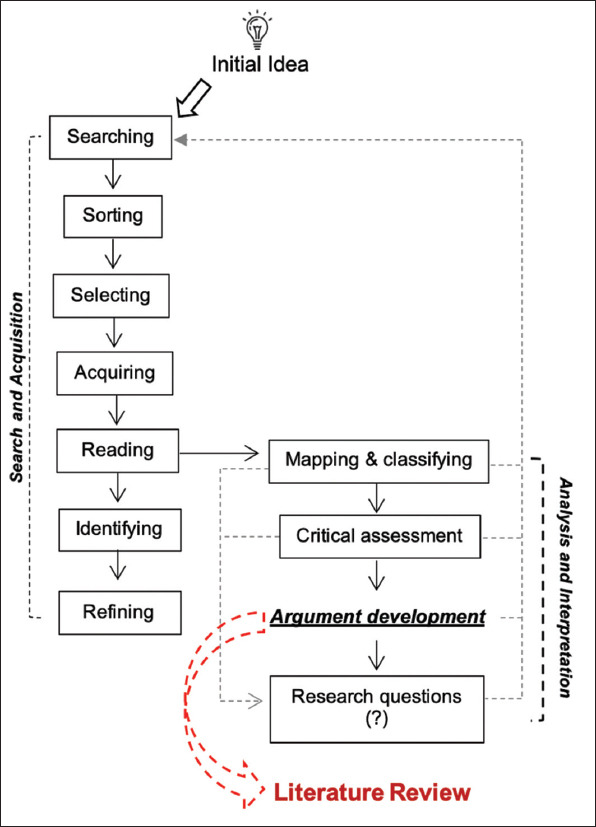
Schematic representation of a hermeneutic framework which consists of two intertwined circles. (Adapted from Boell SK *et al*.) [12]

After consulting a librarian, a hermeneutic systematic approach was applied, since this method suits well the multifaceted subject of the study. The process consisted of: (1) Searching and citation tracking in PubMed and Web of Science to gather articles on angiogenesis in vascular malformations, and (2) analysis and interpretation of the articles on potential factors inducing MVP in the lesions (Figure 2). This iterative process aims to deepen understanding of the subject. Searching is systematic but versatile, allowing relevant articles to be critically interpreted and ideas to be understood in the context of the subject. The process of understanding should be seen as open ended and circular in nature. A conventional systematic review has a highly structured search strategy and consequently downplays the importance of interaction between the literature and reader. This interaction is of high value as it leads to creative ideas, seeking originality rather than reproducibility. Searching together with reading interchangeably encircled relevant articles which provided valuable information. Database searching, citation tracking and snowballing have been used to gather high value articles to answer the research questions [12]. Inclusion and exclusion criteria for the study are mentioned in Table 2.

**Table 2:** Inclusion and exclusion criteria

Inclusion criteria	Exclusion criteria
•Patients (all ages) with congenital vascular malformation in skin and/or soft tissue.	•Malignancy
	•Cardiac
	•Intracranial
•All study designs	•Retinal
	•Pulmonic
	•Other languages than English
	•Syndromes associated with arteriovenous malformations

A study by Meijer-Jorna *et al.*, identifying MVP in AVM for the 1^st^ time, served as initial starting point [4]. A generic search was applied in *PubMed* using keywords for “AVM” and “angiogenesis.” Titles were screened, and relevant articles were selected and used for understanding the background. Through extensive reading, relevant factors involved in AVM expansion could be identified (Table 4). New searches were done in *Web of Science* and *PubMed*, in which the exclusion criteria were added to reduce the number of hits. Still numerous unrelated articles came up, which were excluded afterwards. Adding “soft tissue” or “skin” as search terms limited the results and were left out. Earlier relevant hand-picked articles came up in the search, which showed only small number of publications regarding angiogenesis in AVM expansion. Through snowballing and citation tracking, four additional studies of relevance were included in the study. These consisted of two articles on the role of hormones and one on matrix metalloproteinases in AVM expansion. A study of a new identified angiogenic protein, Angiogenic Factor With G-Patch and FHA Domains 1 (AGGF1) and its expression in AVM tissue was included in the study. Latest update of the search took place during the second revision of this manuscript (May 2021).

**Table 3:** Overview of 16 included studies, quality assessed by Newcastle-Ottawa Scale (NOS)

Author	AVM Samples (n), Mean age, F:M	Main Findings	Quality assessment using NOS			

			S	C	O/E	T
Dawson [24]	14, 39.5 year, 6:8	Absence of a systemic angiogenic factor.	★	★	★	Good
Duyka [27]	12, 41.89 year, 8:4	10 of the 12 AVM samples (83%) stained diffusely positive for PGR compared to no staining in control. (*p*<0.001).	★	★	★	Good
Kulungowski [26]	11, NR, NR	GHR expression was increased in AVM compared to control (*p*=0.01).	★	★	★	Good
Liu [14]	272 p, NR, NR	Progression was more common during adolescence (65%) compared to childhood (38.8%) (*p*=0.002).	★	★	★	Good
Lu [17]	12, 22.4 year, 4:8	Increased MVD in Stage III (5.8%) compared to Stage II (1.3%) (*p*=0.004).	★	★	★	Good
Maclellan [25]	10, NR, NR	FSHR expression was increased in AVM compared to other vascular anomalies (*p*<0.0001).	★	★	★	Good
Marler [15]	25p, 16.5 year, 10:15	Increased urinary hMW (125 kd) MMPs in extensive and unremitting AVM 100% (*p*=0.01).	★	★	★	Good
Meijer-Jorna [4]	71, 25 year, 16:16	32 cases of MVP of which 30 (94%) AVM (*p*<0.001).	★	★	★	Good
Meijer-Jorna [16]	80, 32 year, 38:42	81% of HFAVM (*n*=37) showed proliferation vs. 14% of LFAVM (*p*<0.005).	★	★	★	Good
Meijer-Jorna [28]	10, NR, NR	5 cases showed multifocal distinct areas of immature capillary vessels.	★	★	★	Good
Pavlov [18]	7, 44.5 year, 4:3	Increased expression of VEGF and VEGFR2 in recurrent AVM compared to primary AVM (*p*<0.05) (*p*>0.05).	★	★	★	Good
Pavlov [19]	7, 44.5 year, 4:3	Elevated proliferation in small vessels (2-4%) compared to adjacent medium sized vessels (1%).	★	★	★	Good
Redondo [11]	1, 51, NA	Increased levels of VEGF (2x) MMP-9 (2x) Ang-2 (10x) Tie-2 (3x) compared to control (*n*=10).	★	★	★	Fair
Ryu [22]	6, 28.3 year, 3:3	Increased gene expression of Ang-2 in EC on AVM compared to normal vascular tissue.	★	★	★	Good
Wautier [23]	2, 22.75 year, 2:2	Increased proliferation compared to control (*p*<0.001).	★	★	★	Fair
Zhan [29]	22, 28 year, 12:10	21 of 22 cases showed AGGF1 expression in plump EC compared to no expression in flat cells (*p*<0.01).	★	★	★	Fair

F:M Female to Male ratio, NR: Not Reported, NA: Not Applicable, MVD= Microvessel density, HFAVM: High flow AVM, LFAVM: Low flow AVM, AGGF1: Angiogenic factor with G-patch and FHA domain 1, hMW: High molecular weight, S: Selection, C: Comparability, O: Outcome, E: Exposure (used for case control) T: Total score

**Table 4:** Summary of 16 included articles

Subject	Type of studies
Angiogenesis in AVM	Histological studies [4,16-19,23,28,29], Retrospective cohort [14], Case control [15,22,24], Case study [11]
Angiogenesis and Hormonal influences	Histological studies [25-27]
Effect of treatment strategies	Histological studies [18,28], Retrospective cohort [14]

**Table 5:** Angiogenic factors reported in AVM skin and soft tissue

Angiogenic factors	References
AGGF-1	Zhan *et al*. [29]
Ang-1	Meijer-Jorna *et al*. [16]
Ang-2	Meijer-Jorna *et al*. [16]
	Redondo *et al*. [11]
	Ryu *et al*. [22]
HIF-1α	Lu *et al*. [17]
Neuropilin	Lu *et al*. [17]
MMP-9	Wei *et al*. [43]
	Redondo *et al*. [11]
Tie-2	Redondo *et al*. [11]
TNF-α	Sainson *et al*. [40]
VEGF	Meijer-Jorna *et al*. [16]
	Lu *et al*. [17]

The quality assessment for all the included articles was performed by two investigators (AMU and SA) independently using Newcastle-Ottawa Scale (NOS). NOS is a straightforward and objective scoring system and resulted in consensus between both investigators. This scoring system awards a star for meeting pre-defined criteria for each of three categories: *The selection of the study groups*, *the comparability*, and *the ascertainment of either the exposure or outcome of interest*, depending on the type of study. A good quality is given when it met 3 or 4 stars in selection domain, 1 or 2 stars in comparability domain, and 2 or 3 stars in outcome/exposure domain, a fair quality was given for 2 stars in selection domain, 1 or 2 stars in comparability domain, and 2 or 3 stars in outcome/exposure domain and a poor quality was given when 0 or 1 star in selection domain, or 0 stars in comparability domain, or 0 or 1 stars in outcome/exposure domain [13].

## 3. Results

Hermeneutic article selection (Figure 2) resulted in 16 articles dealing with MVP in AVM (Table 3). They were further divided into three sub-subjects: Occurrence of angiogenesis in AVM based on histological and/or biological features, angiogenesis, and hormonal influences, and anti-angiogenic effect of the treatment strategies. The types of studies were histological studies, retrospective cohort, case control, and case study (Table 4). The search terms and the flow chart are presented in the supplementary data 1 and 2. The summary (author, study population, research question, method, statistical analysis, and main results) of these articles are presented in the supplementary data 3, where the NOS quality assessment analysis in supplementary data 4.

### 3.1. Angiogenesis in AVM

The 16 articles listed in Table 3 reported various parameters of vasoproliferative growth. Histologically, AVM are composed of large and tortuous arteries with reactive intimal changes and large and thick-walled veins, accompanied by smaller thin-walled vessels. Meijer-Jorna *et al*. found additional proliferations composed of closely packed microvessels with plump endothelium (immature microvessels) in between the large pre-existent vessels in a series of vascular malformations (*n*=32), of which 94% were AVM and 6% were venous malformation (*n*=2), and which were predominantly located in the head and neck regions. Interestingly, the extent of vasoproliferative growth was reported to be significantly more abundant in males than in females [4]. Other histological parameters of MVP reported in the articles were: increased mast cell (MC) density [3], micro vessel density (MVD) [3,10,12], and Ki-67 (cellular marker for proliferation) immunostaining [3,10-14]. Clinical studies were based on sudden onset growth in existing vascular malformations, which was interpreted by the authors as angiogenesis or MVP [14,15].

Vascular endothelial growth factor (VEGF), VEGF-A, and VEGF receptors (VEGFR and VEGFR2) expression was reported to be increased in the microvascular proliferative areas compared to surrounding mature vessels [16-19]. Rothbart *et al*. found that all patients exhibited VEGF expression in AVM lesions. No difference in VEGF expression was found in arteries and veins in these AVM lesions [20]. An observational analytic study reported that the majority (30 out of 34 AVM patients) showed VEGF expression [21]. This was also reported in a study showing expression of mRNA expression of VEGF-A, in AVM lesions in both proliferative and non-proliferative areas [16,20]. However, *in situ*, overall expression of VEGF-A and Ang-1 was higher in proliferative areas compared to mature areas. Another angiogenic factor that was reported to in AVM tissue is β-Fibroblast Growth Factor (β-FGF) [20,21]. The Angiopoetin (Ang)/Tyrosin kinase receptor-2 (Tie-2) pathway was also reported in studies on angiogenesis in AVM lesions, especially the increased expression of Ang-2. Tie-2 expression was similar in microvessels compared to mature vessels. Ang-2 was not expressed in either immature or mature vessels. Nevertheless, a previous study reported increased expression of Ang-2 in lesional EC of AVM lesion compared to normal vascular tissue [16,22]. Increased levels of transforming growth factor beta (TGF-β) but not β-FGF were found in cultured AVM EC [19].

In proliferation assays of cultured AVM EC interleukin-1β (IL-1β), tumor necrosis factor-alfa (TNF-α), interferon gamma (IFN-γ) and TGF-β, had no influence on [Methyl-^3^H] thymidine incorporation in AVM. Quantitative measurement of [Methyl-^3^H] thymidine incorporation in cultured cells is widely used as an indicator of cell proliferation. There was no expression of adhesion molecules E-selectin/CD62 and VCAM-1/CD106 after IL-1β and TNF-α stimulation, whereas ICAM-1/CD51 was increased 6 to 15-fold compared to control [23]. The presence of a circulating systemic angiogenic factor in the sera of 14 patients was tested using an assay which successfully demonstrated such activity in diabetics with proliferative retinopathy. However, the assay failed to detect a circulating systemic factor in the AVM group [24]. On the contrary, a case study by Redondo *et al*. on a patient with vascular proliferation in AVM reported increased serum levels of VEGF and matrix metalloproteinases (MMP)-9 (2-fold), Ang-2 (10-fold), Tie-2 (3-fold) compared to control sera (*n*=10), and decreased levels of platelet derived growth factor (PDGF), PDGF-AB, and PDGF-BB [11].

### 3.2. Angiogenesis and hormonal influences

The presence of hormone receptors has also been reported in AVM. Expression of follicle-stimulating hormone (FSH), androgen (A), estrogen (E), progesterone (P), and growth hormone (GH) receptors was tested (FSHR, AR, ER, PGR, and GHR, respectively) [25-27]. FSHR expression was increased in AVM compared to other types of vascular malformations (*p*<0.0001). There was no expression in control tissue. Furthermore, GHR was highly increased in AVM tissue (72.7% compared to controls 25.8%; *p*=0.01). When present, receptor density was similar between AVM and control. Patients with a clinical Schobinger Stage III AVM had increased GHR compared to Stage II lesions (*p*=0.05). Age, sex, and location had no effect on GHR expression (*p=*0.8). Expression of ER, AR, and PGR, which assumed to be responsible in AVM expansion, did not differ compared to control (*p*=0.2) [26].

Interestingly, another study reported that 10 of the 12 (83%) AVM samples showed diffuse positive immunohistochemical staining for PGR compared to no expression in control tissue (*p*<0.0001) [27]. There was no expression of ER in any of the samples.

## 4. Discussion

Although it is well known that AVM of skin and soft tissue gradually expand over time, the underlying mechanisms and pathological features are incompletely understood. The examined data in this review clearly suggest involvement of angiogenesis as can be understood from the histopathological identification of masses of proliferating microvessels in at least part of the reported lesions. In addition, the identification of multiple angiogenic factors in the sera or tissues of AVM patients could support this view. These findings could explain the episodes of abnormal sudden growth accompanied by discomfort up to serious complications in patients. This is further endorsed by the publications on patients with proliferative features in AVM who also showed a high Schobinger stage of clinical severity of the disease.

### 4.1. Angiogenesis in AVM

Angiogenesis is regulated by the balance between pro- and anti-angiogenic factors. A certain disbalance could therefore drive vascular proliferation in AVM, potentially leading to lesion expansion [5]. Growth factors and angiogenic cytokines, such as VEGF and IL-8, are reported to have an important role in the pathogenesis of cerebral AVMs [30]. The present review showed that the same conditions can also be found in the development of AVMs in skin and connective tissue. MC can serve as a source of growth factors. A number of cytokines and angiogenesis-inducing growth factors are produced by the substantially increased numbers of MC that have been reported in areas of MVP of soft-tissue AVMs and skin [4].

The VEGF family of growth factors include the most important stimulators of EC migration and sprouting [31,32]. This implies that VEGF can be important in the pathogenesis of AVM due to stimulating MVP, and likely with the involvement of Ang-1 and Ang-2 [33-35]. Ang-1 is usually found to be elevated in immature micro-vessels when compared to mature micro-vessels [16]. Simple EC will mature toward complete vascular structures due to Ang-1/Tie-2 activity. In contrast, Ang-2 competitively inhibits the activity of Ang-1-induced Tie-2. In the vascular network of AVM, an imbalance in the Ang-Tie-2 system is reported, in the form of increased Ang-2 expression, and decreased in Ang-1 expression, possibly interfering with Tie-2 expression. Inhibitions of the Ang-1 signal, due to Tie-2 expression, this will result in vascular deconstruction, characterized by dilation of blood vessels in the absence of a mature endothelial support structure [22,36,37]. An up to 10 times increase of Ang-2 concentration, as has been found in the AVM, triggers Tie-2 phosphorylation and may cause vascular instability. Furthermore, the decreased expression of Tie-2 as found in AVM can further worsen vascular stability [16,37]. Considering the similarities of AVM in brains and of skin and soft tissue, the Ang-Tie-2 pathway growth factors appear to be involved in skin and connective tissue AVMs. In addition, VEGF also increases upregulation of several other growth factors, [38] and among these increased expression of TGF-β has been reported in EC of AVM [19].

Little is known about the involvement of FGF in AVM expansion. They are nonetheless potent regulators of cell proliferation, differentiation, and function and could therefore be studied to identify their possible role in disturbed vessel growth present in AVM of skin and soft tissue [39]. FGF was found to stimulate VEGF expression in vascular smooth muscle cells (SMC) and has a role in modifying fibroblasts that will form cerebral AVM lesions. In AVM lesions, this FGF is reported to be expressed in perivascular tissue and tunica media [21].

TNF-α is a cytokine also thought to be involved in angiogenesis. TNF-α is pro-angiogenic *in vivo*, but promotes apoptosis *in vitro* [40]. In cultured AVMs exposed to IL-1β, IFN-γ, TGF-β and TNF-α. Intercellular adhesion molecule (ICAM) expression was highly increased, but E-selectin and vascular cell adhesion molecule (VCAM) were not expressed. The apparent dysregulation of leukocyte adhesion molecules expression may pose a barrier to leukocyte infiltration, thus inhibiting local inflammation (as a potential contributor to angiogenesis) [23].

Recently, a new angiogenic and anti-inflammatory agent, AGGF1, was reportedt o be highly expressed in activated EC and MC of AVM. The phophatidylinositol 3-kinase (PI3K) pathway is a regulator of cell growth and dysregulation of this pathway could support the proliferation of EC and disruption of vasculogenesis. Mutation of phosphatidylinositol 3-kinase subunits (PIK3CA), the gene encoding the subunit of PI3K, is associated with AGGF1 expression and will result in the dysregulation [29,41,42]. AGGF1 has also been detected in vascular tumors [29], so further evaluation of its role in MVP of AVM will be of interest.

### 4.2. Angiogenesis in relation to the clinical manifestations of disease

In a retrospective cohort study on natural progression and recurrence of extra cranial AVM, involving 272 individuals, 43.8% of the lesions progressed before adolescence, 82.6% before adulthood and 17.5% worsened at the age of adulthood. Diffuse AVM tended to progress more quickly, in both childhood and adolescence, compared to localized lesions (*p*<0.001). Also 18 pregnancies in 12 women were reported with untreated AVM stage I (*n*=11) and stage II (*n*=1), of which 44.4% led to progression of AVM to a higher Schobinger stage. However, there was no risk difference compared to non-pregnant women (*p*=0.20) [14].

A study by Meijer-Jorna *et al*. found 50% of the proliferative foci to be multicentric and of which 38% appeared to have a solid growth patterns splitting up surrounding adipose and skeletal tissues. Patients in these series underwent surgical resection of lesion because of symptoms (pain, swelling and growth) [4]. It was speculated that MVP plays a part in the onset of symptoms. Thus, occurring in clinically higher staged AVM. In the higher Schobinger Stages II and II of extracranial AVM, increased rates of endothelial progenitor cells (EPC) and vasculogenic factors have been reported, which may further endorse the role of MVP in lesion progression [17]. CD43^+^CD133^+^ EPC were increased in stage III AVM (0.53%) compared to stage II (0.25%) (*p*=0.02). The expression of the vasculogenic factors stromal derived factor (SDF-1α) and hypoxia inducible factor (HIF-α) gene were determined by quantitative real-time reverse-transcriptase polymerase chain reaction. They were increased in Stage III (7.9-fold) compared to Stage II (3.3-fold) (*p*=0.02) and Stage III (7.6-fold) to Stage II (1.7-fold) (*p*=0.02), respectively. Expression of VEGF in Stage II and Stage III was similar (*p*=0.7). However, expression of VEGFR2, Neuropilin 1 and Neuropilin 2 was found to be increased in Stage II compared to Stage III (*p*=0.03). Taken together, these reported findings suggest that MVP, at least episodically, could lead to increase of the lesional mass of AVM.

### 4.3. Hormonal influences

Patients with AVM have the highest risk of progression to a higher Schobinger stage in adolescence suggesting that circulating hormones might contribute to AVM proliferation [14,25]. GH is known to be a major regulator of linear postnatal growth and peaks during puberty [44]. GH acts as a pro-angiogenic factor inducing EC proliferation, migration and formation of capillaries *in vitro* [45,46].

Abnormal expression of GH was found in the endothelium and vascular SMC of AVM. GH could directly or indirectly, for example, through promotion of expression and activity of endothelial citric oxide synthase, be involved in AVM expansion [33,45].

FSH surges during adolescence and their receptor expression is reported to be elevated in AVM. Unlike GH, FSHR are not expressed on normal tissue [25,47]. FSH/FSHR has been identified in several cancer tissues and is supposed to play a role in angiogenesis [47]. However, this still needs to be validated. The role of FSHR in AVM progression is only speculative at this time.

None of the included studies found an association between sex, localization or age, and proliferation, with exception of Meijer-Jorna *et al.*, who found significantly higher proliferative (Ki-67) indexes of lesional EC and SMC in male patients than in female patients. The tissue extent of proliferation appeared also to be more prominent in males [4]. Although not proven as yet, these finding could relate to hormonal effects [4]. Testosterone can directly and indirectly influence angiogenesis. These effects are sex-specific and probably partially due to reduced AR expression in women [48]. Estrogens activate gene regulation through their receptors and consequently enhance the pathophysiological processes of angiogenesis in EC. Both ER and testosterone receptors were reported to be weakly expressed in AVM samples [26]. This does not preclude their possible role in AVM expansion since they can stimulate cell proliferation indirectly through VEGF and other pathways [48,49].

Progesterone is not involved in the onset of puberty or in the transition to adulthood, the timespan in which AVM tend to progress [50]. The reported presence of PGR in lesions could therefore imply that progesterone will be a less potent endothelial mitogen compared to the other hormones. As mentioned earlier, pregnant women with Stage I lesions do not have an increased risk of progression compared to non-pregnant women [14]. Overall, involvement of hormonal influences in AVM expansion can be anticipated, but due to the relative scarcity of data, further investigations are clearly needed.

### 4.4. Hypoxia, inflammatory, and hemodynamic mediated angiogenesis

There is evidence that inflammation is a contributing factor in the pathology of cerebral AVM [51], and potentially the same applies for AVM of skin and soft tissue. In resection samples of extracranial AVM, Meijer-Jorna *et al*. observed more extensive chronic inflammation in high-flow lesions than in low-flow lesions, although in this study no significant differences were observed between the high flow lesions with or without proliferating microvessels. The authors concluded that this might be due to the small sample sizes of at least part of the study materials [28]. However, their finding could also imply that inflammation is not a fundamental factor in the onset of expansion in skin and soft tissue AVM [14].

MMPs regulate several functions related to inflammation, including activity and bioavailability of inflammatory cytokines and chemokines [52]. MMP levels were found to be elevated in patients with AVM, in the form of hMW MMP (125 kDa). This MMP form is a complex of MMP-9 and neutrophil gelatinase-associated lipocalin (NGAL) [15,43]. MMP-9 has the ability to degrade vascular extracellular components including collagen types IV and V, fibronectin, and elastin, and. increased levels of MMP-9 are often found in structurally unstable vessels [37]. MMPs have also been reported in extracranial AVMs [53], which could explain the absence of a number of extracellular components in the AVM lesion [21]. MMP-9 is often secreted by inflammatory cells, especially neutrophils and macrophages, which could endorse the assumption that expansion of extracranial AVMs is associated with an inflammatory process. The discovery of neutrophils in lesions also explains the presence of NGAL in AVM of skin and connective tissues. NGAL is a 25 kDa glycoprotein secreted by neutrophils and binds to MMP-9 to form MMP-9/NGAL complexes [43]. Increased urinary levels of MMP-9/NGAL complexes were also found at least in patients with cerebral AVM [54]. It is suggested that this complex might be a feature especially of large vascular malformations.

Hypoxia is a potent inducer of VEGF. The Hypoxia-Inducible Factor (HIF) pathway contributes to the regulation of both physiologic and pathologic vascular wall remodeling [55], and activation of this pathway is an important stimulus for the growth of blood vessels in tumors. HIF-1α and HIF-2α regulate the expression of VEGF, Ang-1, Ang-2, and Tie-2 [53]. HIF-1α is expressed in all nucleated cells whereas HIF2-α is strictly expressed in a number of cells, including vascular EC [55]. Significantly increased levels of HIF-1α were reported in higher staged extracranial AVM compared to lower staged cases, suggesting that there is a progression of disease due to hypoxic conditions [17]. HIF-1α expression has been reported also in cerebral AVM [56,57]. In experimental studies on brains of mice, a pro-angiogenic state with up regulation of HIF-1α and its downstream targets was found as a result of venous hypertension due to AVM shunting [58]. Increased levels of HIF-1α and HIF-2α have also been observed in gastrointestinal vascular malformations [59]. It is unclear whether increased HIF levels are protective or disruptive. Initially, the HIF pathway will likely be activated to protect the vascular wall cells from the hemodynamic stress, but on the long term, the turbulent blood flow, and altered shear stress due to AVM shunting can lead to pathologic remodeling of the blood vessels and stimulate sprouting of new vessels [28,55]. In resected AVM tissues, this remodeling can be identified histologically as tortuosity of arteries, intimal proliferations, and arterialization of veins. Inflammation, hemodynamic stress, and hypoxia clearly relate to each other and were also reported to be likely involved in post-embolization induced MVP [60].

### 4.5. Angiogenesis: A reactive process or a feature inherent to the lesion

Several suggestions have been made regarding the nature of neovascularization in AVM. Absence of a systemic angiogenic effect may indicate that the factors influencing proliferation are active locally. Cultured AVM cells showed a higher spontaneous proliferation rate compared to human umbilical vein, arterial, or microvascular EC. The proliferation rate of the AVM cells was not responding to inhibitory activity. These findings suggest that the high propensity to proliferate might be caused by an inherent defect [24]. Embryonic arteriovenous shunts may fail to undergo apoptosis and contribute to the presence of AVM [6]. In contrast, pyogenic granuloma, diffuse dermal angiomatosis, and acro-angiodermatitis are all reactive capillary lesions, which occur in response to acquired stimuli such as inflammation trauma or hypoxia/ischemia. Since they also show, at least episodically, the histomorphology and immunophenotypic features of MVP, it could be that microvascular proliferative activity in AVM also represents a reactive process as well [4]. As stated above, reactive process such as tissue hypoxia and inflammatory cells have reported to be a strong driving forces for angiogenesis [53].

### 4.6. Genetics involvement

Recently, several studies have unraveled a number of genetic mutations in AVM. Mutation of the mitogen-activated protein kinase 1 (MAP2K1) gene on EC was assumed to affect EC function and the initiation of pathological arteriovenous shunting through signaling activation of RAS/mitogen-activated protein kinase (RAS/MAPK). This aberration was also presumed to promote angiogenesis [61]. Activated Kirsten-Rat sarcoma 2 viral oncogene homolog (KRAS) gene mutations were found in a proportion of patients with brain AVM [62,63]. Nikolaev *et al*. identified a KRAS mutation in cerebral AVM lesions that coincided with dysregulation of the MAPK-extracellular-signal-regulated kinase (MAPK-ERK) pathway, which is also associated with the development of a number of types of cancer [62]. The MAPK-ERK signaling pathway is considered as an alternative pathway capable of inducing IL-8 and VEGF expression which are major factors promoting angiogenesis [63,64].

HHT is a rare condition characterized by telangiectasia and congenital AF, and is caused by mutations in endoglin/CD105 and activin receptor-like kinase-1 (ALK1) [65,66]. Endoglin knockdown in mice affected the VEGF-A mediated VEGFR2 kinetics and promoted protein kinase B (AKT) signaling, which resulted in survival and vascular growth in response to extracellular signals [66]. Erythropoietin-producing hepatoma receptor B4 (EPHB4) is a transmembrane receptor from the tyrosine kinase family with membrane expression on venous EC. Activation of EPHB4 will reduce ERK phosphorylation and EC proliferation, while inhibition of EPHB4 causes activation of ERK1/2 and triggers angiogenesis. The stop mutation in EPHB4 was found in CAVM cases which could explain the occurrence of angiogenesis in CAVM. RASA1 is an advanced effector or EPHB4 in EC. Mutations in RASA1 and EPHB4 are sometimes found together in cases of CAVM [65,67,68].

### 4.7. Effect of treatment strategies

It is known that AVM can recur and expand following treatment. One study found levels of VEGF and VEGFR to be increased in recurrent AVM tissues when compared to primary AVM tissues. Average VEGF and VEGFR concentrations in primary AVM were 4.80±1.34 pg/mg protein and 61.80±20.85 pg/mg (*p*<0.05) compared to recurrent AVM 21.50±0.27 pg/mg and 545±243 pg/mg (*p*>0.05) [18].

In a retrospective cohort study, recurrence of AVM after intervention was 93%, during a follow-up of 8.9±5.2 years. The types of treatment, being embolization and resection (with or without embolization), were both independently prognostic for recurrence (*p*<0.001), whereas age, location, sex, and size of the lesion were not (*p*>0.05). Resection (with or without embolization) of the AVM had a lower re-expansion rate (81%) compared to treatment by embolization (98%). Embolization alone showed a higher risk of return 14.2 times (95% CI: 4.7-42.2) compared to resection (with or without embolization) (*p*<0.001). Lower staged AVM also had a lower recurrence rate (*p*<0.001). Resection (with or without embolization) also extended the period to clinically significant recurrence of the lesion; 57.3% re-expanded <1 year compared to 85.6% after embolization only (*p*<0.001). Multiple resections or embolization did not decrease the rate of recurrence (*p*=0.59) [14].

Since VEGF is recognized for its important role in the pathology of skin and soft tissue AVMs, inhibition of VEGF could be used as an adjuvant therapy after primary therapy (surgery or radiosurgery). This approach might be able to suppress the proliferation process and accelerate the process of decreasing microvessels density. In addition, several other anti-angiogenesis-based may be useful in the treatment of AVM. A currently common anti-angiogenic therapy in oncology is the application of bevacizumab, a monoclonal antibody that binds to VEGF-A [69]. Mutations in the KRAS and MAPK-ERK pathways which can occur in AVM of skin and soft tissue as stated earlier, could serve as a target for treatment using the currently available MEK inhibitors, Trametinib, or Cobimetinib. A successful study reported that Trametinib therapy was able to reduce the volume of AVM after 6 months [70].

## 5. Conclusion

This systematic review clearly supports the involvement of angiogenesis in the expansion of vascular malformations, and particularly in the subgroup of AVM. The studies confirmed that active angiogenesis is correlated with a higher Schobinger Stage in AVM progression. Expansion of AVM can have detrimental effects on both physical and mental wellbeing of patients, reducing the quality of life. Since there is no cure yet and recurrence rate after treatment is high for AVM, further research is therefore required to better understand the angiogenic microproliferative processes in AVM. This could serve to develop novel anti-angiogenic pharmacotherapy. Targeted pharmacotherapy directed to any of the pathways outlined in this review, may have therapeutic potential. This review is limited by the scarce amount of existing literature regarding angiogenesis in AVM of skin and soft tissue.*In vitro* and *in vivo* studies are necessary to further unravel which angiogenic mechanisms are involved and whether anti-angiogenic agents could have a significant role to inhibit vasoproliferation in AVM of skin and soft tissue. A large prospective cohort study is proposed to provide more insights in the relationship between vasoproliferation and clinical symptoms.

## Figures and Tables

**Supplementary Data 1:**
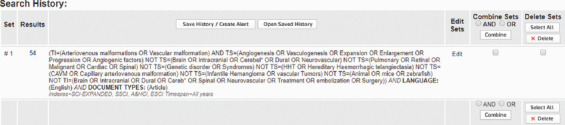
Flow chart. **Search terms: *Web of Science***

**Supplementary Data 2:**
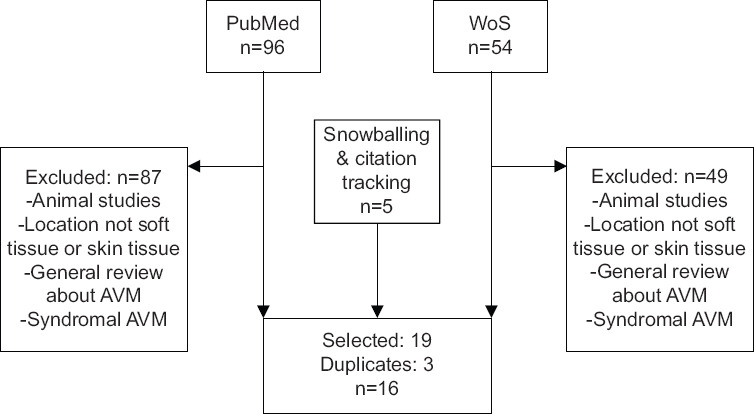
Flow chart.

## Appendix

A literature review of microvascular proliferation in arteriovenous malformations of skin and soft tissue

(TI=(Arteriovenous malformations OR Vascular malformation) AND TS=(Angiogenesis OR Vasculogenesis OR Expansion OR Enlargement OR Progression OR Angiogenic factors) NOT TS=(Brain OR Intracranial OR Cerebel* OR Dural OR Neurovascular) NOT TS=(Pulmonary OR Retinal OR Malignant OR Cardiac OR Spinal) NOT TS=(Genetic disorder OR Syndromes) NOT TS=(HHT OR Hereditary Haemorrhagic telangiectasia) NOT TS=(CAVM OR Capillary arteriovenous malformation) NOT TS=(Infantile Hemangioma OR vascular Tumors) NOT TS=(Animal OR mice OR zebrafish) NOT TI=(Brain OR Intracranial OR Dural OR Cereb* OR Spinal OR Neurovascular OR Treatment OR embolization OR Surgery)) AND LANGUAGE:(English) AND DOCUMENT TYPES: (Article) Indexes=SCI-EXPANDED, SSCI, A and HCI, ESCI Timespan=All years

**Search terms: *PubMed***

(((Arteriovenous malformations [tiab] AND (angiogenesis [tiab] OR enlargement [tiab] OR proliferation [tiab] OR expansion [tiab] OR vasculogenesis [tiab] OR Angiogenic [protein])) NOT (intracranial[tiab] OR brain [tiab] OR cerebral[tiab] OR spinal [tiab]OR retinal[tiab] OR dural[tiab] OR HHT [tiab] OR Hereditary hemorrhagic telangiectasia [tiab] OR CAVM [tiab]) Sort by: Best Match Filters: Humans

**Supplementary Data 3:** Summary of 16 included articles

Author, year	Study population	Research question	Method	Statistical analysis	Main results and notes
Dawson *et al*. 1993	14 patients 8:6 M:F 25–54 years	Are there angiogenic factors found in the blood of patients with AVM?	Blood samples 14 patients with high flow AVM	Multiple means comparison and t- test	No circulating angiogenic factor in the small group patients with AVM. No significant difference between AVM group and controls *p*=0.31 Sample size too small, they calculated that 215 patients were at least needed. This might indicate that the factors influencing growth and development of these lesions do so locally and a systemic therapeutic approach might not be the solution.
Duyka, 2009	12 people, 8 female, 4 men with AVM.	To identify hormone receptors within vascular malformations (arteriovenous malformations [AVMs], venous malformations [VMs], and lymphatic malformations [LMs]) of the head and neck	12 AVM were stained for both ER and PR. 10 breast carcinoma specimens were used as controls and endothelium and smooth muscle cells of the blood vessels serving as negative controls.	Fisher exact test was used for statistical analysis.	Ten of the 12 (83%) AVM specimens stained diffusely positive for PR within the nuclei of the endothelium and smooth muscle of the malformed vessels (*p*<0.0001). None of the vascular malformation specimens stained positive for ER.
Jorna *et al.*2006	107 specimens of skin/soft tissue AVM=71 Retrospective	Aim: to systematically investigate the presence and extent of microvascular proliferation in a large series of surgically treated VMs	KI-67/CD31 KI67/SMA-1	Binary logistic regression models, paired *t*-test, Mann-Whitney U tests, Fischer’s exact test	Areas of microvascular proliferation were found amid the mature vessel of the malformation in 32 out of 107 cases. 30 cases were AVM. 42% of AVM showed microvascular proliferation. MVD and MCD significantly higher in areas of microvascular proliferation. Both EC and SMC showed high Ki-67 labeling indexes. Proliferative activity predominantly in vessels <20µm Ki-67 labeling indexes in both EC and SMC and extent of microvascular proliferation were higher in male patient than female. Probably relate to a shorter cell cycle in male VSMCs than in those from female. Pain, swelling, rapid growth, and/or functional impairment were symptoms most frequently encountered in this population, serving as indicator for surgery. It could be that microvascular proliferation is involved in causing symptoms due to mass-forming effect.
Jorna et al. 2012	80 AVM	Investigate the relationship between a microvascular proliferative response and flow velocity in AVM	Reviewing samples for info on flow and assessment anti-CD31, Sma-1. Inflammation: Anti CD3, HLA-DR, anti-CD79a, anti-CD68 and anti-Tryptase Thrombosis: blood cloth presence Vascular leakage: vWF CD105	Ax^2^test was used	Clusters or even diffuse sheet like patterns. Specific for immature microvascular growth, were found in 30 of 37 high flow cases and 6 of 43 low flow cases. In the embolized group of AVM, 21 of 24 showed areas of microvascular proliferation. 1 low flow was embolized and showed no proliferation. In the non-embolized group proliferation was found in 27% of the cases 9 (69%) high flow and 6 (14%) low-flow In 13 cases with a record of previous surgery, 7/13 (54%) showed microvascular growth compared with 29 of 67 (43%) in AVM of patients operated on for the first time. Inflammatory infiltrates noticed in 25 of 37 (68%) high flow AVM and 15 of 43 (35%) low flow *p*<0.05 composed of diffusely spread macrophages and mast cells Found in 21 of 24 embolized high flow lesions CD105 present in 84% and not in normal skin. Mainly in areas of microvascular proliferation and venous component. These findings suggest a clear relationship between flow forces inside the malformation and angiogenesis of microvessels. The fistulas → fast flow, flow turbulences and altered shear stress in vascular bed → angiogenesis. Formation of microvascular sprouts has received much attention. Because they occur only episodically and can only be recognized histopathologically and only become manifest in a subset of AVM. → if arelation exists between symptoms and proliferation, pharmacotherapy might have potential.
Jorna et al. 2012	10 AVM 5 VM 8 IH 5 PG	Presence of vasoproliferative foci, conglomerates of matured microvessels and compared microvascular proliferation areas	Anti-CD31, anti SMA-1 VEFG-A, Ang-1 Ki-67, p16, p21, p27, Active caspase-3, P53	Mean standard deviation. Mann- Whitney U test.	All AVM samples showed multifocal distinct areas of immature capillary vessels. mRNA of Ang-1 Ang-2 and VEGF-A could be detected in both proliferative and non-proliferative areas in Vascular malformations. Thus, no clear difference in presence of angiogenic proteins between both groups. Immunoreactivity of angiogenic proteins appeared significantly higher in immature than mature areas with exception of Ang-2. AVM showed a microvascular maturation pattern similar to IH and PG. AVM showed similar transitional pattern as seen in PG and IH Areas high of Ki-67 also showed high levels of p16. Angiogenesis cannot explain all the disproportionate growth of symptomatic AVM; formation of collaterals, dilatation and thickening of veins or inflammation and ulceration all cause enlargement too.
Kulungowski et al. 2011	Prospective 54 patients AVM *n*=11	Determine the presence of receptors for testosterone, estrogen, progesterone and/or growth hormone. Also, whether their expression differs from age sex and location matched control tissues.	Immunohistochemistry; staining for hormone receptors.	Chi-square, Wilcoxon rank test. Binary logistic regression. Multivariate analysis	Growth hormone receptor was more commonly found in AVM 72.2% compared to control 25.7 and *p*=0.01. Was only located in endothelium/smooth muscle cells 62.5% or both endothelium/smooth muscle cells and stroma (37.5%) Age sec and location did not affect the presence ofgrowth hormone receptor *p*=0.08. Schobinger Stage III AVM were more likely to express Growth hormone receptor (8/9) compared to Stage II lesions (0/2) *p*=0.05. Expression of androgen, estrogen, and progesterone receptor did not differ from control specimen. Odds ratio growth hormonereceptor expression in AVM is 7.7 compared to control. p: 0.010 Growth hormone is produced steadily during childhood and then peaks during puberty. This correlated with the clinical observation that vascular malformations may expand during childhood and risk increased 2–2.6-fold during adolescence. Suggests that Growth Hormone which has proangiogenic activity may affect to be less likely than growth hormone to influence VM. Estrogen and testosterone might affect expansion indirectly, for example, they stimulate growth hormone production and signaling.
Liu *et al*. 2009	272 patients	To determine the timing of AVM progression and its recurrence rate after intervention.	Follow up patients. Clinical data sampling.	Chi square, Fishers, Binary logistic regression.	All children with stage I AVM had progression of their lesion. 43.8 and before adolescence and 82.6% before adulthood. Progression was more common during adolescence. Mean age at risk for progression was 12.7 + 11.1 years. Multiple regression events were also greater in adolescence compared to childhood. Sex and location did not affect rate of progression; however, diffuse AVM were more likely to progress compared to localized lesions *p*<0.001. 18 pregnancies occurred in 12 women with Stage I (*n*=11) and stage 2 (*n*=1) who were not previously treated. AVM progressed to a higher Schobinger stage in 8 (44.4%) of the pregnancies which did not differ from the risk of AVM progression in non-pregnant women (*p*=0.20) Recurrence after intervention was documented in 93.0% of patients. Average follow up was 8.9 + 5.2 years. Multivariate analysis showed that the type of treatment (embolization vs. resection with or without embolization) and stage at treatment I-IV were both independent predictors of recurrence *p*<0.001. However, sex (*p*=0.10), Location (*p*=0.60) size (*p*=0.07) and age (*p*=0.21) did not correlate with recurrence. Resection (with or without embolization) re-expansion rate=81 Embolization alone re-expansion rate=98% Recurrence was less n lower stages lesions (*p*<0.001) Patients manages with embolization alone had a 14.2 (95% CI 4.7–42.2) times greater risk of recurrence compared with patient treated with resection (with or without embolization) *p*<0.001 Resection also prolonged the time to recurrence. 57.3% re-expanded within 1 year of intervention compared with 85.6% managed with embolization alone *p*<0.001. Repetition of embolization or resection. did not reduce recurrence rate
Lu L.	12 specimens from 12 patients prospectively mean age: 22.4 years Did not mention sex.	Determine if more severe AVMs exhibit increased angiogenic/vasculogenic factors compared to lower-staged lesions. Comparison stage II (*n*=7) to stage 3 (*n*=5)	CD31, Ki67, CD34/CD133 and RT-PCR: VEGF, SDF-1a, HIF-α VEGFR1 VEGFR2 Neuropilin 1,2 MVD counted 2 blinded people Compared to control tissue Unclear who’s tissueLesions were pre-embolized	Unpaired t test or one-way ANOVA. *p*<0.05 considered significant	↑ CD31 in Stg. 3 – Stg. 2 *p*=0.004 #proliferating EC in Stage 3 similar to Stage 2: *p*=0.08 ↑ CD34, CD133 EPC 2.1 x Stg. II – III; *p*=0.02 Ki67 index similar in Stage II and Stage III → suggests that neovascularization not results from angiogenesis ↑ SDF-1α Stg. 3 (7.9x) – Stg. 2 (3.3) *p*=0.02 ↑ HIF-1α Stg. 3 (7.6x) – Stg. 2 (1.7) *p*=0.02 mRNA VEGF similar. Functional VEGF receptor elevated in Stg.3 NRP1 and NRP2 Stg.3 (5.8) and (4.6) Stg. 2 (3.0) and (2.4) *P*=0.03 Vasculogenesis rather than angiogenesis might be responsible for expansion
Maclellan *et al*. 2013	AVM (*n*=10) No mentioning of sex	To determine whether vascular anomalies express FSH receptor and compare FSH receptor expression in vascular anomalies to to other vascular tumors and control tissues	Immunofluorescence: FSH, MVD	Chi square, Fisher’s exact. Wilcoxon rank-sum test	The expression of FSH was elevated in AVM (2.65) percent compared with CM (1.02), VM (0.76) and LM (0.38) (*p*<0.0001) FSH has been implicated in the pathogenesis of cancer and has also been shown to induce neovascularization by increasing HIF-1a in target tissues and promotes VEGF signaling FSH expression correlates with the clinical behavior of vascular anomalies. It can be hypothesized that FSH is involved in the pathogenesis of vascular malformations.
Marler *et al*. 2005	217 with vascular anomalies 25 AVM F/M: 10/15	To determine whether urinary MMP’s bFGF and VEGF are detectable in patients with vascular tumors and malformations. And to evaluate whether expression is increased as a function of disease extent and progression.	Substrate gel electrophoresis. Enzyme-linked immunosorbent assay.	Stratification Pearson x2 Kruskal–Wallis and Mann-Whitney U test. Fisher. Multistepwise logistic regression.	hMW MMP’s were increased in the urine of pt with vascular malformations (41% ; *p*=0.005) Abnormal bFGF levels high in vascular malformations (27% *p*=0.002) hMW MMP’s significantly increased in patients with extensive and unremitting AVM (100% *p*=0.01) The 125-kd band of MMP’s was elevated in the AVM group. 125-kd is a complex of MMP-9and NGAL (neutrophil gelatinase-associated lipocalin). They demonstrated that complexing of NGAL with MMP-9 protects MMP-9 from autodegradation. This has been found in the urine of adults with metastatic cancer. Suggest that this complex is a feature of extensive and unremitting vascular malformations. Increased levels of MPP-9 have been found in adult patients with brain AVM.
Pavlov *et al* 2009	7 AVM F:M 4:3	Studies the expression of angiogenic mediators in endotheliocytes and the proliferative activity of these cells in AVM and VM	Staining for: VEGF, TGF-β3 b-FGF and Ki-67		Moderately elevated expression of TGF-β In endotheliocytes in both VM and AVM. Higher in AVM Low expression of β-FGF in VM and AVM Endotheliocyte proliferation was low (0.1%) but moderately elevated in the small proliferative vessels (2-4%) VEGF higher in AVM than VM The growth of cultured endotheliocytes from AVM does not change after addition of TGF-β while the growth of normal endotheliocytes is inhibited by this factor and their apoptosis is induced. In addition, a moderate and in some cases a significant increase of expression TGFb1 and its ALK5 receptor and their matrix RNA by endotheliocytes of the cerebral AVM was detected. TGF-β is probably involved in the development and progress of AVM. 3 samples showed high TGF-β and the others moderate expression.
Pavlov *et al.* 2009	13 pt F:M 7:6 22-67 years AVM *n*=7 4:3	Comparing the expression or concentration of VEGF-A and VEGFR2 in tissue samples of Arteriovenous and venous malformations	Immunohistochemical study and enzyme immunoassay		Immunohistochemical study of AVM and VM revealed a moderate increase of VEGF. Expression in endotheliocytes in AVM was higher. (very strong in 3/7 samples) The reaction was more pronounced in samples from recurrent forms of AVM. VEGF concentration was low in primary AVM 3.8–5.7 pg/mg protein. Average content 4.80 pg/mg protein. Concentration was higher in recurrent AVM 21.3–21.7 pg/mg protein average content 21.50 *p*<0.05 Content of VEGFR2 in primary AVM average: 61.80 ± 20.86 pg/mg protein was lower than in recurrent AVM average: 545 ± 243 pg/mg protein *p*>0.05.
Redondo, 2007	Case report of a 51- year old man with etensive arteriovenous vascular malformation in the trunk.		Serum angiogenic factors of the patiënt were compared to control subjects (*n*=10)		At the age of 20 years he underwent an amputation of his left arm because of incoercible repeated hemorrhagic episodes. Since then, the lesion has progressively grown, and soft, large, circumscribed blue-black tumors that repeatedly bleed have appeared. In our patient, we found increased Ang-2 levels, which also occur in some brain arteriovenous malformations. We also detected increased levels of Tie-2 soluble receptor. Angiopoientin 2 is predominantly expressed in areas undergoing vascular remodeling. This could suggest that there is an abnormal disassembly level between endothelial cells and mesenchymal cells due to an abnormal balance in the Ang-2–Tie-2 system, leading to dilated vessels with insufficient mural cell components. Vascular endothelial growth factor (VEGF) and matrix metalloproteinase 9 (MMP-9) serum levels (increased ×2), angiopoientin 2 (Ang-2) levels (increased ×10), and Tie-2 (receptor tyrosine kinase-2) levels (increased ×3) were increased in comparison to the control group. Platelet-derived growth factor (PDGF) AB (PDGF-AB) and PDGF-BB levels were decreased (in one-third of the control group). The presence of an imbalance of angiogenic factors in this patient is in favor of their role in the pathogenesis of at least some vascular malformations.
Ryu JY *et al* (2021)	Case control study for patients with AVM	To determine shear stress in angiogenesis process in AVM	Measuring gene expression of angiogenic factor before and after given shear stress in AVM and normal vascular tissue.	Paired and unpaired t-test	Gene expression of Ang-2 and TGFβR1 were increased in EC of AVM, with or without shear stress, compared to normal vascular tissue.
Wautier et al. 1999	Initial 11 but only 4 suitable for culture and only 2 for analysis.	Goal was to attempt to culture endothelial cells from AVM and characterize these cells according to antigen expression, rate of proliferation and response to cytokines.	Il-1b, TNF-α, IFN-y TGF-β	Mean standard deviation Wilcoxon rank sum test for paired values and one-way ANOVA and Dunnett’s test.	Proliferation of AVM was higher than of Human umbilical vein endothelial cells or human microvascular endothelial cells.yz IL-1B, TNF-α, IFN-γ and TGF-β did not alter AVM proliferation. Neither E-selectin nor VCAM-1 was detectable on AVM surface after stimulation with IL-1β or TNF-α. Constitutive ICAM-1/CD51 expression was 6–15-fold higher than observed in other lesions. The non-expression of leukocyte adhesion molecules may represent a border to lymphocyte-monocyte infiltration and constitute a resistance to the defense mechanism mediated by leukocytes. A factor secreted by local tissue cannot be responsible for the down response to cytokines because the lack of response was observed after several weeks of culture. They speculate that some of the growth regulation alteration may be secondary to the abnormal flow conditions. However, the abnormal control of proliferation remained in culture. Ets-1 expression suggest reduction of apoptosis process Suggest an intrinsic defect.
Wautier *et al.* 1999	Initial 11 but only 4 suitable for culture and only 2 for analysis.	Goal was to attempt to culture endothelial cells from AVM and characterize these cells according to antigen expression, rate of proliferation and response to cytokines.	Il-1b, TNF-α, IFN-y TGF-β	Mean standard deviation Wilcoxon Rank Sum Test for Paired values and One-way ANOVA and Dunnett’s test.	Proliferation of AVM was higher than of Human umbilical vein endothelial cells or human microvascular endothelial cells. IL-1α, TNF-α, IFN-γ and TGF-β did not alter AVM proliferation. Neither E-selectin nor VCAM-1 was detectable on AVM surface after stimulation with IL-1β or TNF-α. Constitutive ICAM-1/CD51 expression was 6–15-fold higher than observed in other lesions. The non-expression of leukocyte adhesion molecules may represent a border to lymphocyte-monocyte infiltration and constitute a resistance to the defense mechanism mediated by leukocytes. A factor secreted by local tissue cannot be responsible for the down response to cytokines because the lack of response was observed after several weeks of culture. They speculate that some of the growth regulation alteration may be secondary to the abnormal flow conditions. However, the abnormal control of proliferation remained in culture. Ets-1 expression suggest reduction of apoptosis process suggest an intrinsic defect.
Zhan *et al.* 2016	119 AVM=22	Investigate AGGF1 expression in VM	Immunohistochemistry anti-AGGF1	Chi square test	Of the 22 cases 21 Cases showed AGGF1 expression in plump endothelial cells. Only 2 of 16 flat endothelial cells showed AGGF1 expression. These findings indicate that preferential expression of AGGF1 in plump endothelial cells was applicable to AVM. Most mast cells also showed AGGF1. Did not correlate to the AGGF1 positive plump endothelial cells. Endothelial cells become plump when activated. AGGF1 expression was examined in HMC-1 cells. Strong granular staining was observed, indicating AGGF1 was contained in mast cell granules. Some parts had undetectable expression. AGGF1 is not specific for VM because vascular tumors also expressed AGGF1.

**Supplementary Data 4:** Newcastle-Ottawa Scale quality assessment analysis

Article	Selection	Comparability	Exposure	Total stars
Meijer-Jorna [4]	1b, 2a 3a 4a	1a, age sex and location	1a 2a 3a	3
Meijer- Jorna [16]	1b 2a 3a 4a	1a demographic data, location, flow analysis, and prior treatment	1a 2a 3a	3
Meijer- Jorna [28]	1b 2a 3a 4a	1a cases and controls same person,	1a 2a 3a	3
Lu [117]	1a 2a 3a 4a	Patients characteristics unclear	1a 2a 3a	3
Zhan [29]	1a 2a 3a 4a	Patients characteristics somewhat similar previous surgery not mentioned cases and controls same person	1d 2a 3a	3
Pavlov [18]	1a 2a 3a 4a	Case and control same person, similar characteristics, and recurrence recorded	1a 2a 3a	3
Pavlov [19]	1a 2a 3a 4a	Case and control same person,	1a 2a 3a	3
Wautier [23]	1a 2b 3a 4a	Small number of cultured cells (only from two patients)	1a 2a 3c	3
Liu [17]	1a 2a 3a 4a	1a	1b 2a(28y) 3a	3
Marler [15]	1a 2b 3a 4	1a	1a 2a	3
Kulungowski [26]	1a 2a 3a 4a	Age sex location type of anomaly	1a 2a 3a	3
Maclellan [25]	1a 2a 3a 4a	Age sex location type of anomaly	1a 2a 3a	3
Dawson [24]	1a 2a 3a 4a	1a	1a 2a 3a	3
Duyka [27]	1a 2a 3a 4a	1a	1a 2a 3a	3
Redondo [11]	1a 2a 3c 4c	1a	1a 2a 3 NA	3
Ryu [22]	1a 2a 3b 4a	Case–Control	1a 2a 3a	3

## References

[1] Mulliken JB, Glowacki J (1982). Hemangiomas and Vascular Malformations in Infants and Children. Plast Reconstr Surg.

[2] Enjolras O (1997). Classification and Management of the Various Superficial Vascular Anomalies:Hemangiomas and Vascular Malformations. J Dermatol.

[3] Greene AK, Liu AS, Mulliken JB, Chalache K, Fishman SJ (2011). Vascular Anomalies in 5,621 Patients:Guidelines for Referral. J Pediatr Surg.

[4] Meijer-Jorna LB, van der Loos CM, de Boer OJ, van der Horst CM, van der Wal AC (2007). Microvascular Proliferation in Congenital Vascular Malformations of Skin and Soft Tissue. J Clin Pathol.

[5] Mcinnes RR (2003). Developmental Biology:Frontiers for Clinical Genetics Vascular Malformations:Localized Defects in Vascular Morphogenesis. J Cell Physiol.

[6] Greene AK, Orbach DB (2011). Management of ARTERIOVENOUS malformations. Clin Plast Surg.

[7] Finn MC, Glowacki J, Mulliken JB (1983). Congenital Vascular Lesions:Clinical Application of a New Classification. J Pediatr Surg.

[8] Merrow AC, Gupta A, Patel MN, Adams DM (2016). 2014 Revised Classification of Vascular Lesions from the International Society for the Study of Vascular Anomalies:Radiologic-Pathologic Update. Radiographics.

[9] International Society for the Study of Vascular Anomalies (ISSVA) (2018). ISSVA Classification for Vascular Anomalies.

[10] Wassef M, Blei F, Adams D, Alomari A, Baselga E, Berenstein A (2015). Vascular Anomalies Classification:Recommendations from the International Society for the Study of Vascular Anomalies. Pediatrics.

[11] Redondo P, Martínez-Cuesta A, Quetglas EG, Idoate M (2007). Active Angiogenesis in an Extensive Arteriovenous Vascular Malformation:A Possible Therapeutic Target?. Arch Dermatol.

[12] Boell SK, Cecez-Kecmanovic D (2010). Literature Reviews and the Hermeneutic Circle. Aust Acad Res Libr.

[13] Wells GA, O'Connell D, Peterson J, Welch V, Losos M, Tugwell P (2014). Newcastle-Ottawa Quality Assessment Scale.

[14] Liu AS, Mulliken JB, Zurakowski D, Fishman SJ, Greene AK (2010). Extracranial Arteriovenous Malformations:Natural Progression and Recurrence after Treatment. Plast Reconstr Surg.

[15] Marler JJ, Fishman SJ, Kilroy SM, Fang J, Upton J, Mulliken JB (2005). Increased Expression of Urinary Matrix Metalloproteinases Parallels the Extent and Activity of Vascular Anomalies. Pediatrics.

[16] Meijer-Jorna LB, van der Loos CM, Teeling P, de Boer OJ, Florquin S, van der Horst CM (2012). Proliferation and Maturation of Microvessels in Arteriovenous Malformations-Expression Patterns of Angiogenic and Cell Cycle-Dependent Factors. J Cutan Pathol.

[17] Lu L, Bischoff J, Mulliken JB, Bielenberg DR, Fishman SJ, Greene AK (2011). Increased Endothelial Progenitor Cells and Vasculogenic Factors in Higher-Staged Arteriovenous Malformations. Plast Reconstr Surg.

[18] Pavlov KA, Gershtein ES, Dubova EA, Shchegolev AI (2011). Vascular endothelial growth factor and Type 2 receptor for this factor in vascular malformations. Bull Exp Biol Med.

[19] Pavlov KA, Dubova EA, Shchyogolev AI, Mishnyov OD (2009). Expression of Growth Factors in Endotheliocytes in Vascular Malformations. Bull Exp Biol Med.

[20] Rothbart D, Awad IA, Lee J, Kim J, Harbaugh R, Criscuolo GR (1996). Expression of Angiogenic Factors and Structural Proteins in Central Nervous System Vascular Malformations. Neurosurgery.

[21] Kiliç T, Pamir N, Küllü S, Eren F, Ozek MM, Black PM (2000). Expression of Structural Proteins and Angiogenic Factors in Cerebrovascular Anomalies. Neurosurgery.

[22] Ryu JY, Kim YH, Lee JS, Lee JW, Oh EJ, Kim HM (2021). Oscillatory Shear Stress Promotes Angiogenic Effects in Arteriovenous Malformations Endothelial Cells. Mol Med.

[23] Wautier MP, Boval B, Chappey O, Enjolras O, Wernert N, Merland JJ (1999). Cultured Endothelial Cells from Human Arteriovenous Malformations Have Defective Growth Regulation. Blood.

[24] Dawson P, Kennedy A, Petty RG (1993). Absence of an Angiogenic Factor in Large Systemic Arteriovenous Malformation. Invest Radiol.

[25] Maclellan RA, Vivero MP, Purcell P, Purcell P, Kozakewich HP, DiVasta AD (2014). Expression of Follicle-Stimulating Hormone Receptor in Vascular Anomalies. Plast Reconstr Surg.

[26] Kulungowski AM, Hassanein AH, Nosé V, Fishman SJ, Mulliken JB, Upton J (2012). Expression of Androgen, Estrogen, Progesterone, and Growth Hormone Receptors in Vascular Malformations. Plast Reconstr Surg.

[27] Duyka LJ, Fan CY, Coviello-Malle JM, Buckmiller L, Suen JY (2009). Progesterone Receptors identified in Vascular Malformations of the Head and Neck. Otolaryngol Head Neck Surg.

[28] Meijer-Jorna LB, van der Loos CM, de Boer OJ, Horrevoets AJ, Mekkes JR, van der Horst CM (2013). Microvascular Proliferations in Arteriovenous Malformations Relate to High-Flow Characteristics, Inflammation, and Previous Therapeutic Embolization of the Lesion. J Am Acad Dermatol.

[29] Zhan M, Hori Y, Wada N, Ikeda J, Hata Y, Osuga K (2016). Angiogenic Factor with G-Patch and FHA Domain 1 (AGGF1) Expression in Human Vascular Lesions. Acta Histochem Cytochem.

[30] Timbang MR, Richter GT (2020). Update on Extracranial Arteriovenous Malformations:A Staged Multidisciplinary Approach. Semin Pediatr Surg.

[31] Kubis N, Levy BI (2004). Understanding Angiogenesis:A Clue for Understanding Vascular Malformations. J Neuroradiol.

[32] Gerhardt H, Golding M, Fruttiger M, Ruhrberg C, Lundkvist A, Abramsson A (2003). VEGF Guides Angiogenic Sprouting Utilizing Endothelial Tip Cell Filopodia. J Cell Biol.

[33] Chen GZ, Ke Y, Qin K, Dong MQ, Zeng SJ, Lin XF (2017). Analysis of the Expression of Angioarchitecture-Related Factors in Patients with Cerebral Arteriovenous Malformation. Chin Med J (Engl).

[34] Koizumi T, Shiraishi T, Hagihara N, Tabuchi K, Hayashi T, Kawano T (2002). Expression of Vascular Endothelial Growth Factors and their Receptors in and Around Intracranial Arteriovenous Malformations. Neurosurgery.

[35] Sure U, Butz N, Schlegel J, Siegel AM, Wakat JP, Mennel HD (2001). Endothelial Proliferation, Neoangiogenesis, and Potential *De Novo* Generation of Cerebrovascular Malformations. J Neurosurg.

[36] Hashimoto T, Lam T, Boudreau NJ, Bollen AW, Lawton MT, Young WL (2001). Abnormal Balance in the Angiopoietin-Tie2 System in Human Brain Arteriovenous Malformations. Circ Res.

[37] Hashimoto T, Young WL (2004). Roles of Angiogenesis and Vascular Remodeling in Brain Vascular Malformations. Semin Cerebrovasc Dis Stroke.

[38] Pulkkinen HH, Kiema M, Lappalainen JP, Toropainen A, Beter M, Tirronen A (2021). BMP6/TAZ-Hippo Signaling Modulates Angiogenesis and Endothelial cell Response to VEGF. Angiogenesis.

[39] Mancini ML, Terzic A, Conley BA, Oxburgh LH, Nicola T, Vary CP (2009). Endoglin Plays Distinct Roles in Vascular Smooth Muscle Cell Recruitment and Regulation of Arteriovenous Identity During Angiogenesis. Dev Dyn.

[40] Sainson RCA, Johnston DA, Chu HC, Holderfield MT, Nakatsu MN, Crampton SP (2008). TNF Primes Endothelial Cells for Angiogenic Sprouting by Inducing a Tip Cell Phenotype. Blood.

[41] Zhang T, Yao Y, Wang J, Li Y, He P, Pasupuleti V (2016). Haploinsufficiency of Klippel-Trenaunay Syndrome gene Aggf1 Inhibits Developmental and Pathological Angiogenesis by Inactivating PI3K and AKT and Disrupts Vascular Integrity by Activating VE-Cadherin. Hum Mol Genet.

[42] Castel P, Carmona FJ, Grego-Bessa J, Berger MF, Viale A, Anderson KV (2016). Somatic PIK3CA Mutations as a Driver of Sporadic Venous Malformations. Sci Transl Med.

[43] Wei T, Zhang H, Cetin N, Miller E, Moak T, Suen JY (2016). Elevated Expression of Matrix Metalloproteinase-9 not Matrix Metalloproteinase-2 Contributes to Progression of Extracranial Arteriovenous Malformation. Sci Rep.

[44] Cianfarani S (2014). Is High-Dose Growth Hormone Treatment during Puberty Worthwhile?. Horm Res Paediatr.

[45] Clapp C, Thebault S, Jeziorski MC, Martínez De La Escalera G (2009). Peptide Hormone Regulation of Angiogenesis. Physiol Rev.

[46] de Lima CF, dos Santos Reis MD, da Silva Ramos FW, Ayres-Martins S, Smaniotto S (2017). Growth Hormone Modulates *In Vitro* Endothelial Cell Migration and Formation of Capillary-Like Structures. Cell Biol Int.

[47] Papadimitriou K, Kountourakis P, Kottorou AE, Antonacopoulou AG, Rolfo C, Peeters M (2016). Follicle-Stimulating Hormone Receptor (FSHR):A Promising Tool in Oncology?. Mol Diagn Ther.

[48] Sieveking DP, Lim P, Chow RW, Dunn LL, Bao S, McGrath KC (2010). A Sex-Specific Role for Androgens in Angiogenesis. J Exp Med.

[49] Losordo DW, Isner JM (2001). Estrogen and Angiogenesis:A Review. Arterioscler Thromb Vasc Biol.

[50] Lee PA, Xenakis T, Winer J, Matsenbaugh S (1976). Puberty in Girls:Correlation of Serum Levels of Gonadotropins, Prolactin, Androgens, Estrogens, and Progestins with Physical Changes. J Clin Endocrinol Metab.

[51] Chen Y, Zhu W, Bollen AW, Lawton MT, Barbaro NM, Dowd CF (2008). Evidence of Inflammatory Cell Involvement in Brain Arteriovenous Malformations. Neurosurgery.

[52] Nissinen L, Kähäri VM (2014). Matrix Metalloproteinases in Inflammation. Biochim Biophys Acta.

[53] Krock BL, Skuli N, Simon MC (2011). Hypoxia-Induced Angiogenesis:Good and Evil. Genes Cancer.

[54] Hashimoto T, Wen G, Lawton MT, Boudreau NJ, Bollen AW, Yang GY (2003). Abnormal Expression of Matrix Metalloproteinases and Tissue Inhibitors of Metalloproteinases in Brain Arteriovenous Malformations. Stroke.

[55] Lim CS, Kiriakidis S, Sandison A, Paleolog EM, Davies AH (2013). Hypoxia-Inducible Factor Pathway and Diseases of the Vascular Wall. J Vasc Surg.

[56] Takagi Y, Kikuta K, Moriwaki T, Aoki T, Nozaki K, Hashimoto N (2011). Expression of Thioredoxin-1 and Hypoxia Inducible Factor-α in Cerebral Arteriovenous Malformations:Possible Role of Redox Regulatory Factor in Neoangiogenic Property. Surg Neurol Int.

[57] Ng I, Tan WL, Ng PY, Lim J (2005). Hypoxia Inducible Factor-1 Alpha and Expression of Vascular Endothelial Growth factor and its Receptors in Cerebral Arteriovenous Malformations. J Clin Neurosci.

[58] Gao P, Zhu Y, Ling F, Shen F, Lee B, Gabriel RA (2009). Nonischemic Cerebral Venous Hypertension Promotes a Pro-Angiogenic Stage through HIF-1 Downstream Genes and Leukocyte-Derived MMP-9. J Cereb Blood Flow Metab.

[59] Tan HH, Ge ZZ, Gao YJ, Chen HM, Fang JY, Chen HY (2011). The role of HIF-1, Angiopoietin-2, Dll4 and Notch1 in Bleeding Gastrointestinal Vascular Malformations and Thalidomide-Associated Actions:A Pilot *In Vivo* Study. J Dig Dis.

[60] Buell TJ, Ding D, Starke RM, Webster Crowley R, Liu KC (2014). Embolization-Induced Angiogenesis in Cerebral Arteriovenous Malformations. J Clin Neurosci.

[61] Smits PJ, Konczyk DJ, Sudduth CL, Goss JA, Greene AK (2020). Endothelial MAP2K1 Mutations in Arteriovenous Malformation Activate the RAS/MAPK Pathway. Biochem Biophys Res Commun.

[62] Nikolaev SI, Vetiska S, Bonilla X, Boudreau E, Jauhiainen S, Rezai Jahromi B (2018). Somatic Activating KRAS Mutations in Arteriovenous Malformations of the Brain. N Engl J Med.

[63] Priemer DS, Vortmeyer AO, Zhang S, Chang HY, Curless KL, Cheng L (2019). Activating KRAS Mutations in Arteriovenous Malformations of the Brain:Frequency and Clinicopathologic Correlation. Hum Pathol.

[64] Guo Y, Pan W, Liu S, Shen ZF, Xu Y, Hu LL (2020). ERK/MAPK Signalling Pathway and Tumorigenesis (Review). Exp Ther Med.

[65] Boon LM, Ballieux F, Vikkula M (2011). Pathogenesis of Vascular Anomalies. Clin Plast Surg.

[66] Jin Y, Muhl L, Burmakin M, Wang Y, Duchez AC, Betsholtz C (2017). Endoglin Prevents Vascular Malformation by Regulating Flow-Induced Cell Migration and Specification Through VEGFR2 Signalling. Nat Cell Biol.

[67] Revencu N, Boon LM, Mendola A, Cordisco MR, Dubois J, Clapuyt P (2013). RASA1 Mutations and Associated Phenotypes in 68 Families with Capillary Malformation-Arteriovenous Malformation. Hum Mutat.

[68] Li Y, Chen H (2020). Novel EPHB4 Mutation in Capillary Malformation-Arteriovenous Malformation Syndrome 2 (CM-AVM2):The First Genetic Study in Asians.

[69] Vernimmen FJ (2014). Vascular Endothelial Growth Factor Blockade:A Potential New Therapy in the Management of Cerebral Arteriovenous Malformations. J Med Hypotheses Ideas.

[70] Ota T, Komiyama M (2020). Pathogenesis of Non-Hereditary Brain Arteriovenous Malformation and Therapeutic Implications. Interv Neuroradiol.

